# Mutual Avoidance in the Spectacled Salamander and Centipede: A Discrepancy between Exploratory Field and Laboratory Data

**DOI:** 10.3390/ani13203214

**Published:** 2023-10-14

**Authors:** Francesco Cerini, Claudio Pardo, Davide Taurozzi, Benedetta Gambioli, Leonardo Vignoli

**Affiliations:** 1 Dipartimento Scienze Ecologiche e Biologiche, Università della Tuscia, 01100 Viterbo, Italy; 2 Dipartimento di Scienze, Università Roma Tre, 00154 Rome, Italy; davide.taurozzi@uniroma3.it (D.T.); benedetta.gambioli@uniroma3.it (B.G.); leonardo.vignoli@uniroma3.it (L.V.)

**Keywords:** *Salamandrina perspicillata*, *Scolopendra cingulata*, co-occurrence, centipede, spectacled salamander, shelter choice, manipulative experiments

## Abstract

**Simple Summary:**

Despite adult amphibians often being predators of arthropods, there are cases where the roles are reversed. This study focuses on the potential predator–prey relationship between the spectacled salamander (*Salamandrina perspicillata*) and the centipede *Scolopendra cingulata* in Central Italy. In a natural site, we observed a strong avoidance pattern (negative co-occurrence) of the two study species under shelters (stones). Given the importance of olfactory cues in the salamander’s behavioural choices, we hypothesized that this species would actively avoid shelters used by the centipede so as to evade potential attacks and predation. However, when we forced the study species to choose between sharing or not sharing a given shelter in a laboratory experiment, the avoidance pattern was not confirmed. Our exploratory results show that what often appears to be a strong observation-based pattern in natural settings needs to be carefully evaluated, and experiments in controlled environments could help exclude potentially misleading processes.

**Abstract:**

Interactions between amphibians and arthropods encompass a wide range of ecological relationships, predominantly characterized by predator–prey dynamics, with adult amphibians as the predators. In some instances, the roles are reversed. This study focuses on the potential predator-prey relationship between the spectacled salamander (*Salamandrina perspicillata*) and the centipede *Scolopendra cingulata* in Central Italy. Building upon previous research on chemical cue perception in amphibians, we investigated potential olfactory cue-mediated avoidance behaviours exhibited by *S. perspicillata* towards the potential predator *S. cingulata* through field observations and manipulative experiments. In a natural site, we estimated the degree of negative co-occurrence between the study species under shelters and found an avoidance pattern between *S. perspicillata* and *S. cingulata* in refuges. However, when the study species were forced to choose between sharing or not sharing a given shelter, through a manipulative experiment, the avoidance pattern was not confirmed. Potential determinants contributing to the avoidance pattern observed in nature are discussed. Our exploratory results represent a good example of how what often appears to be a strong observation-based pattern in natural settings needs to be carefully scrutinized. Hypotheses testing through experiments in controlled environments remains a valuable approach to exclude potentially misleading processes.

## 1. Introduction

Amphibians and arthropods interact in a wide range of forms in nature, encompassing intra-guild competition for space and food as well as mutual predation [1,2]. Generally, predation happens with the amphibians exploiting arthropods as their main food resource [3,4]. However, cases in which this relationship is reversed are not uncommon, especially in tropical areas. In fact, venomous arthropods are capable of capturing prey that is both larger and smaller than their own body length [5]. Cases of amphibian predation by arthropods have been widely documented in many regions of the world throughout the years [5,6]. For example, in the tropical forests of Asia and South America, many spiders (e.g., Ctenidae family; [7]), giant water bugs (e.g., Belostomatidae family; [8]) and beetles (e.g., Cicindelidae family; [9]) have been reported as preying upon metamorphosed amphibians. For centipedes (Scolopendridae), predatory acts towards juvenile snakes [7,10] were more often documented than those towards anurans [11]. In the Paleartic region, Odonata nymphs and Dytiscidae beetles [12], among many other aquatic insects, commonly prey upon larval stages in amphibians, but little is known about the role of arthropods as predators of adult amphibians.

*Scolopendra cingulata*, Latreille 1829, like other centipedes, has a modified jaw that injects paralyzing venom into its prey [13]. In the Mediterranean region, most of the knowledge concerning *S. cingulata*’s vertebrate preys involve lizards [14,15], while no evidence of direct predation on amphibians has been reported. In Central Italy, *S. cingulata* coexists syntopically with an endemic salamander species, the spectacled salamander *Salamandrina perspicillata* (Savi 1821), which due to size and habitat use could potentially be subjected to predation. *Salamandrina perspicillata* is a mainly terrestrial amphibian which, besides oviposition activity and larval development, spends most of its life wandering and hiding in the forest litter [16,17]. *Salamandrina perspicillata* shows both diurnal and nocturnal patterns of activity [18] and, similarly to *S. cingulata,* which is mainly nocturnal [19], uses stones and rotting logs as elected cover substrates on the forest floor. Notwithstanding such similar spatial ecology, events of *S. cingulata* directly preying on *S. perspicillata* are not reported in the literature. This might be due to the lack of investigations and the elusiveness of both species. Generally, direct interactions between salamanders and predacious invertebrates are hard to observe in the field and can be challenging for ecologists to comprehend [20]. In fact, urodeles might compete with arthropods for food resources, while both might be subjected to mutual predation in different stages of life [6]. Nevertheless, there are cases of direct attack and predation on juvenile urodeles by spiders [21,22].

*Salamandrina perspicillata* can use olfactory cues to discriminate between home waters and other sites during the breeding season, and to choose the best terrestrial refuge based on the scent of other individuals [23,24]. Generally, this species’ behaviour is influenced by the scent of its conspecifics [24], similar to other species of the order. For example, Eastern Red-backed Salamanders display aggressive behaviour as a reaction to conspecifics, but also to centipedes and beetles [25]. Other urodeles (e.g., *Salamandra salamandra*) can, in the early stages of their life, sense the chemical cues left by a predator and avoid them [26]. We argue that the same mechanisms could be used by *S. perspicillata* to detect the presence or absence of a potential predator in a refuge before entering it. Cases of predation on adult *Salamandrina* are reported only anecdotally (i.e., one event by freshwater crab on a female in water; [27]). In a terrestrial habitat, it can be assumed that wild boars may feed on amphibians, and thus on spectacled salamanders, as occurs in other areas [28]. Conversely, predation by large invertebrates on spectacled salamanders has been largely overlooked in terrestrial habitats and can only be hypothesised; indeed, we cannot rule out that it may occur and have an impact on salamander populations. Thus, the aim of the study was to investigate whether *S. perspicillata* would show an avoidance pattern towards the potential predator *S. cingulata* in selecting shelters. We investigated this potential behaviour by recording the co-occurrence of *S. perspicillata* and *S. cingulata* in refuges both in the field and in manipulative experiments. We expect a negative co-occurrence distribution pattern to emerge if a true antagonistic behaviour (i.e., predation/competition) exists between the study species [29,30]. Manipulative experiments, by standardizing and limiting the availability of refugia, should exacerbate any potential negative interspecific interaction, thus providing stronger evidence of its occurrence.

## 2. Materials and Methods

### 2.1. Study Area and Field Experiments

**Figure 1:**
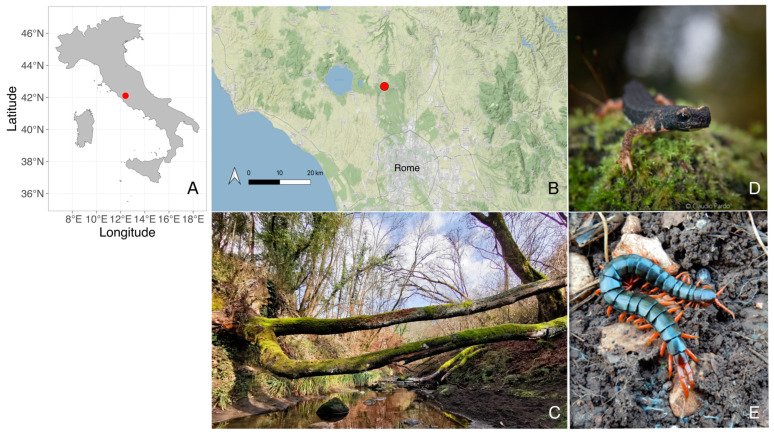
Overview of study site and animals. (**A**) Map of Italy with geographical placement of Vejo Regional Park (red point). (**B**) Zoom on the portion of central Italy where the site is located; the city of Rome is indicated for reference. (**C**) Picture of the habitat typology where the animals were sampled. (**D**) Portrait of *Salamandrina perspicillata.* (**E**) Portrait of *Scolopendra cingulata* (Copyrights: Claudio Pardo for salamander photo; Leonardo Vignoli for habitat photo; https://commons.wikimedia.org/wiki/File:Scolopendra_cingulata_Mt._Carmel.jpg [accessed on 10 June 2023] for centipede photo).

The study was conducted in Vejo Regional Park (42.105° N, 12.405° E; Latium region, Central Italy), a protected area on the northern outskirts of Rome (Figure 1). Samplings were carried out in an oak (*Quercus cerris*) forest surrounding a small effluent of the Crémera river that forms a gorge with steep banks. In the autumn of 2009, sampling was performed for three days (15th and 19th of October, 26th of November), providing the first unpublished data from which the pattern of avoidance between the salamander and the centipede was initially inferred. Ten years later, three surveys were carried out on the 26th and 29th of November and the 10th of December 2019, during the “post courtship” ecological phase, when the animals can be found in feeding activity on land [31]. Consistent with the 2009 protocol, the sampling activity was performed along a 15 m linear transect on the riverbanks, inspecting 22 natural shelters (stones) of a similar size, taking note of the presence and absence of *S. perspicillata* and *S. cingulata* under each shelter.

### 2.2. Manipulative Experiments

**Figure 2:**
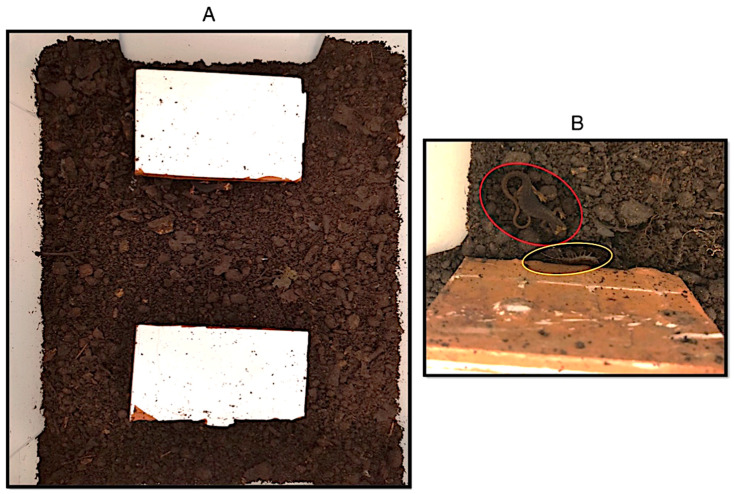
Experiment terrarium set-up. (**A**) Detail of experimental arena, with forest soil as substrate and the two tiles acting as shelters. (**B**) Zoom on a case of proximity of *Salamandrina perspicillata* (red circle) and *Scolopendra cingulata* (yellow circle) at the end of a trial.

In January 2020 we captured 23 individuals of *S. perspicillata* and five individuals of *S. cingulata* in the same study area, and we brought them to the laboratory to perform an experimental test of shelter choice. The animals were housed in two separated plastic “home” terraria (57 × 39 × 28 cm), with leaf litter from the study area as substrate. The experimental protocol consisted of a 3-day procedure: on day 1, an individual of *S. cingulata* was placed in a smaller “test” terrarium (39 × 28 × 14 cm) filled with a thin layer of leaf litter coming from the “home” terraria; this included two identical shelters (6 × 4 cm tiles) placed at opposite sides of the enclosure (Figure 2). This allowed the animal to settle under one of the two shelters. On day 2, one *S. perspicillata* individual was placed in the centre of the “test” terrarium. The room was kept at a constant temperature of 16 °C and 90% relative humidity; artificial lights were programmed to replicate the natural photoperiod. Data on the ability of salamanders to differentiate among chemicals on the substrate suggest that visual cues are not necessary to elicit a response from homo- and hetero-specific signals, even in complete darkness [1,24]. On day 3, the terrarium was checked in the morning, when the lights had been on for two hours, to ensure the animals were under a shelter, and the position of the individuals was recorded. Indeed, both animals are prone to refuge under a shelter to avoid direct contact with the light. After each experiment, the tiles were cleaned using distilled water and sterile tissues to remove any odorous or chemical cues that could have been left. The first set of experiments consisted of 18 trials, where the terrarium substrate was uniformly moistened by means of a nebulizer before and after every test. After the first round of experiments, we observed a low usage of the tiles as shelter by the salamanders. Thus, we performed a second set of 23 trials, with the same protocol, but we moistened just the substrate under the shelters (tiles) to ensure a higher probability of test success (i.e., the two animals using the shelters). In both sets of tests, spectacled salamander individuals were tested just once to avoid pseudo-replication. On the contrary, due to a lack of sampled individuals, the centipedes were tested more than once.

## 3. Statistical Analysis

To analyse the potential avoidance pattern of the two species in the natural sites, we transformed the records of the two species under the transect stones into Sites-x-Species presence/absence matrices, one per each sampling session. The stones under which neither of the two study species were found were not included in the matrices. We performed a probabilistic co-occurrence analysis [32] on such matrices to measure whether the frequency of the two species not sharing a shelter (negative co-occurrence) would be significantly different from a random association. Analyses were performed with R (version 4.3.0, https://cran.r-project.org/ [accessed on 9 October 2023]) using the function cooccur() of the *cooccur* package [33], with the alpha value set at 0.05. 

For the laboratory tests, we considered successful those trials where both *S. perspicillata* and *S. cingulata* were found under the shelters; among the successful trials, we recorded the occurrence of the avoidance (i.e., the two species not sharing the same shelter). We ran a Generalized Linear Model (GLM, binomial distribution, logit link function) using the outcome of the trials (0 = avoidance; 1 = shared shelter) as the dependent variable, and the experimental design (uniform vs. shelter moisture) as a predictor. We considered a statistically significant intercept (i.e., intercept *p*-value of the GLM < 0.05, when the predictor is zero) as indicating that the outcome of the trials was different from a 50% probability of success (i.e., random choice). 

## 4. Results

### 4.1. Field Experiments

The results of the co-occurrence analysis are summarized in Table 1. In one of the three sampling days of 2019 (10th December), no animals were found under the 22 stones (i.e., not included in the table). All the sampling sessions of 2009 resulted in a very significant avoidance pattern (negative association with *p* < 0.005, Table 1). The sampling performed on the 26th of November 2019 had a low number of sites with animal presence, which was not sufficient for a significant statistical power in the classification of the co-occurrence pattern (i.e., unclassifiable, Table 1). The second sampling date of 2019 resulted in a significant negative association.

### 4.2. Manipulative Experiments

**Figure 3:**
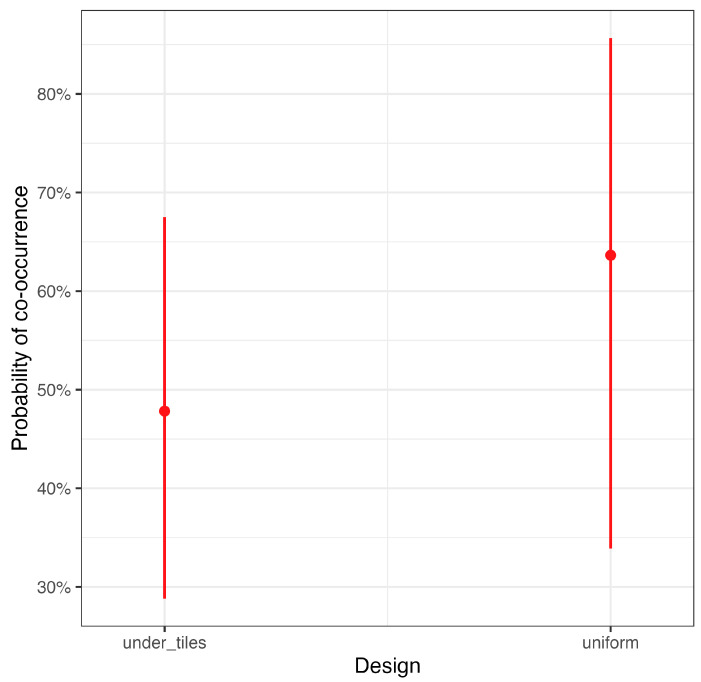
Predicted probability of co-occurrence (mean + 95% CI) from the GLM, comparing the experiment with humidification performed under the tiles vs. the experiment with uniform humidification.

The first experiment, where the terrarium was uniformly humidified, resulted in a low number of successful tests (i.e., animal using the shelters and not being found around the arena after 24 h, Table 2), and salamanders selected the shelter without *S. cingulata* in just four out of eleven trials (36% of cases exhibiting avoidance). The second set of experiments had a 100% success rate of animals using the shelters, but we observed avoidance in about 50% of the tests (Table 2), a frequency not different from a random association. The GLM confirmed the absence of a pattern of avoidance different from chance in both experiments as well as the absence of an effect of the experimental design (i.e., humidification) (Table 3, Figure 3).

## 5. Discussion

Our study reports a potential avoidance behaviour observed in the field between a predatory arthropod and a small salamander. We estimated this behaviour by assessing the degree of negative species co-occurrence under shelters. However, through laboratory manipulative experiments, the avoidance pattern was not confirmed. 

The capacity of amphibians to perform active avoidance behaviour based on predator cues has been demonstrated in different species, particularly anurans [34,35] and especially at the larval stage [29,36,37]. Similarly, in urodeles, evidence of predator avoidance behaviour has been found in some species (e.g., *Salamandra salamandra* [26], *Cryptobranchus alleganiensis* [38]). In particular, *Plethodon cinereus* showed aggressive behaviour when experimentally paired with centipedes [25]. Additionally, juveniles of the same species increased escape times and performed submissive poses when exposed to chemical cues of centipedes, and the two taxa showed a negative co-occurrence pattern both in field and laboratory testing [39]. Although the authors did not find evidence of direct predation, they hypothesized that the ability of the salamanders to detect the centipedes’ chemical cues and to react with behavioural changes might result in decreasing general negative interactions [39]. Therefore, we assumed that such an adaptive skill could have been the cause of the strong pattern we observed in the field sites, where *S. perspicillata* almost never co-occurred with *S. cingulata* under natural shelters. The importance of other olfactory cues in determining behavioural choices in *S. perspicillata* [24] formed a solid basis for our hypothesis. Our laboratory experiments, albeit limited in the number of replicates, suggest that the salamanders do not actively avoid sharing a shelter with the centipedes, despite such species being an aggressive and potential predator of other small vertebrates [14,15]. If the animals were adapted to perceive the chemical or visual cues of *S. cingulata* as a predation risk [38,40], and given the small effort required for a salamander to change shelter (i.e., due to the limited space of the test arenas), we would have expected the avoidance pattern to arise more frequently compared to a random choice. The high frequency of actual co-occurrence of the two species under the same tile might indicate that the centipede does not tend to attack or aggressively interact with the salamander, even in the case of strict physical proximity. This suggests the centipedes’ lack of interest towards *S. perspicillata* as a potential prey or danger. However, such speculation needs to be supported by additional tests. In fact, we conducted the experiments with a small number of *S. cingulata* individuals, potentially introducing uncontrolled factors into the tests, such as repeatedly testing a less aggressive individual or one producing non-repelling chemical cues. Our experimental setting (i.e., very small enclosure with just two shelters) should rule out the possibility that the salamanders miss the encounter with the centipede or its chemical tracks, even in complete darkness [24]. Indeed, it is very unlikely that the studied salamanders would choose a shelter without having the perception of centipede occurrence in the proximity. Therefore, if a negative interaction between the species were to occur, we would expect the salamanders to move from the tile where a centipede is located to look for another tile. Although we observed the salamanders exploring the enclosure before seeking refuge under an available tile, it is possible that, in such a short-lived experiment (24 h), some salamanders might take refuge without fully exploring the entire enclosure and being unaware of the centipede’s presence. Conducting more focused experimental tests on the direct interaction between the study species would help clarify these hypothesized processes.

The pattern of avoidance observed in the field is open to various interpretations, as the negative association arising from the probabilistic co-occurrence analysis can be used to infer habitat preferences, distributional patterns, and interactions between the species involved (e.g., competition or predation [32]). The direct predation of the centipedes on *Salamandrina perspicillata* (i.e., intra-guild predation) in cases of shared natural shelter might generate the negative co-occurrence; indeed, intra-guild predation was found to generate avoidance patterns in insects [41]. The absence of such predation events and of the avoidance pattern in our laboratory tests, although limited in number, make such a hypothesis improbable. Additionally, the avoidance pattern might arise from several negative, but not fatal, interactions (e.g., attempted predation, aggressive displays [20]) between the salamanders and the centipedes that, over time, make the salamander or the centipede avoid the other. In such cases, the previous experience and the age of salamanders might play an important role [26]; however, checking the individuals for previous exposure to the centipede in natural settings is clearly not feasible. Moreover, if the salamander eats the centipede’s youngsters, a similar pattern could occur that might explain the contradictory results found in the laboratory and in nature. However, the few studies exploring the diet of *S. perspicillata* report that the centipedes are a very infrequent prey item (less than 1% of the entire food spectrum [42]), making this hypothesis unlikely. We did not observe a consistency between the use of some of the 22 stones sampled along the transect by any of the two species; thus, we could exclude a segregation emerging from preferential choice of shelters due to different environmental variables. Nevertheless, we did not measure the detailed physical or chemical microhabitat features that could possibly explain the segregation pattern to confirm such inference. Most likely, the avoidance pattern might have emerged due to other random or uncontrolled factors, or simply to the surplus in shelter availability. For example, the immediate surroundings of the transect might have hosted several potentially used recoveries that we did not inspect, such as small rocky dens or dead wood logs. It is possible that the inclusion of other potential unchecked recoveries in the species–sites matrix could have changed the co-occurrence analysis results to reflect the random association found in the experimental tests. 

Generally, integrating field-based hypothesis with laboratory tests, and vice versa, is considered a best practice in science, often yielding promising results. For example, a study on invasive species combined both laboratory and field data to obtain a more accurate assessment of their negative impacts on local communities [43]. Similarly, the hypothesis of predation on invasive fish eggs by native species was initially inferred through field observation and confirmed through experiments [44]. Importantly, potentially negative interactions are often investigated by integrating field and laboratory data, leading to consistent findings between the two approaches, as seen in studies involving freshwater fish [45] and anurans [46]. However, controlled experiments that investigate processes leading to co-occurrence patterns are relatively rare. For instance, in one study, a negative co-occurrence pattern was observed between two dragonfly species in drinking troughs, and it was hypothesized that this resulted from intra-guild predation. Subsequent laboratory aquarium tests confirmed this process as the mechanism generating spatial segregation [41]. Similarly, the negative effects of an invasive frog on native tadpole survival were identified in laboratory experiments, and these findings were consistent with a C-score-based community structure analysis [47]. On the other hand, there are instances in which field surveys do not align with laboratory-based prediction, as seen in a study on the impact of an invasive amphibian on Australian herpetofauna [48]. In our case, the inconsistency between field and laboratory observations led to some considerations that may help explain the different outcomes of the two investigations. First, the field observations, although documenting the natural behaviour of the species without manipulation, could have been influenced by various uncontrolled factors (e.g., parameters that were not considered in the analyses but affected shelter selection by the species). Additionally, the limited number of sampling days might not have allowed a consistent pattern to emerge. Yet, in the controversy between field and laboratory experiments, the opposite is also true: due to oversimplification of the lab experimental setting, differences between data from field and laboratory experiments can be extreme [49]. However, as in this specific case, if the natural availability of resources (i.e., shelters) exceeds the requirements of the animals, the study system is prone to providing an avoidance pattern as a result of mere low occupancy probability: that is, if the number of shelters suitable for occupancy is significantly larger than the number of users, the chance of finding two users under the same shelter is low, regardless of the possible interaction between the users that might influence shelter selection. Indeed, in the manipulative experiments, we significantly reduced the availability of shelters by forcing the animals to select between two tiles, thus avoiding the possible confounding factors related to the shelter surplus characterizing the field experiment.

## 6. Conclusions

This study represents a good example of how what often appears to be a strong observation-based pattern in natural settings needs to be carefully interpreted. Investigating a field-based hypothesis through experimental testing in controlled settings remains a valuable approach to exclude potentially misleading processes. Nonetheless, this study made use of a limited amount of field activity and of experimental replicates; thus, we believe that a more thorough sampling activity performed on larger scale and with a multi-site approach might help clarify the factors that induced the observed avoidance pattern. We believe such exploratory results could foster future studies to explore the ecology of a unique and endemic amphibian species of the Italian peninsula.

## Figures and Tables

**Table 1:** Co-occurrence analysis results of field data sampled in 2009 and 2019. All the classifiable samplings provided a negative (i.e., avoidance) pattern of co-occurrence between the study species. Sampling date: date when the sampling was performed. n Stones: number of stones out of the 22 in the linear transect where either of the two species was found. *S. perspicillata* and *S. cingulata*: number of presences of each species under the shelters. Obs Co-oc: observed co-occurrence value based on the presence–absence matrix. Exp Co-oc: expected co-occurrence value if the two species were distributed randomly. p_lt: probability that the two species would co-occur at a frequency lower than that observed if the two species were distributed randomly. Classification: output of the cooccur() function specifying if the analysed pair of species shows a significant negative association (avoidance).

Sampling Date	n Stones	*S. perspicillata*	*S. cingulata*	Obs Co-oc	Exp Co-oc	p_lt	Classification
15 October 2009	19	11	12	0.366	6.9	0.006	Negative
29 October 2009	12	4	8	0	2.7	0.002	Negative
7 November 2009	16	11	6	1	4.1	0.001	Negative
26 November 2019	6	2	4	0	NA	NA	Unclassifiable
29 November 2019	13	6	8	1	3.7	0.004	Negative

**Table 2:** Experimental test summary. The two sets of experiments (1 and 2) differed in the humidification style of the terrarium before the trials, respectively with water uniformly sprayed on all the substrate or just under the two tiles used as shelters. Total trials: the number of tests (and number of animals tested) in each setting. Success trials: number of tests where the two animals were found under the shelters. Avoidance: number of tests where the animals did not co-occur under the shelters, which, when divided by the success trials, gives the segregation frequency.

Experiment	Humidification	Total Trials	Success Trials	Avoidance	Frequency
1	uniform	18	11	4	0.363
2	under tiles	23	23	12	0.521

**Table 3:** Summary table of the GLM analysing the experiment outcome, with the experiment design (i.e., humidification regime) as the categorical factor. The experiment with moisturization performed under the tiles is used as a baseline. The intercept *p*-value indicates that the intercept estimate is not different from zero and thus not different from a random probability (i.e., 50%) of observing co-occurrence. The outcomes from the experiment with uniform humidification (Coefficient “Design_uniform”) do not differ from those from the other experimental setting or from chance.

Coefficients	Estimate	Std. Error	z Value	*p*
Intercept	−0.087	0.417	−0.208	0.835
Design_uniform	0.646	0.753	0.859	0.391

## Data Availability

Data are currently openly available at the link: https://github.com/franzmatches/S.-perspicillata-S.-cingulata-co-occurrence/tree/main (accessed on 9 October 2023), and are associated to the Zenodo repository https://zenodo.org/record/8434426 (accessed on 9 October 2023).
